# Resource Management in Energy Harvesting Cooperative IoT Network under QoS Constraints

**DOI:** 10.3390/s18103560

**Published:** 2018-10-20

**Authors:** Maliha Amjad, Ashfaq Ahmed, Muhammad Naeem, Muhammad Awais, Waleed Ejaz, Alagan Anpalagan

**Affiliations:** 1 Department of Electrical & Computer Engineering, COMSATS University Islamabad, Wah Campus, Wah Cantonment 47040, Pakistan; malihaamjad@ciitwah.edu.pk (M.A.); ashfaqahmed@ciitwah.edu.pk (A.A.); mnaeem@ciitwah.edu.pk (M.N.); muhammadawais@ciitwah.edu.pk (M.A.); 2 Department of Applied Science & Engineering, Thompson Rivers University (TRU), Kamloops, BC V2C 0C8, Canada; waleed.ejaz@ieee.org; 3 Department of Electrical and Computer Engineering, Ryerson University, Toronto, ON M5B 2K3, Canada

**Keywords:** energy harvesting, IoT, resource management, cooperative communication

## Abstract

Cooperative communication with RF energy harvesting relays has emerged as a promising technique to improve the reliability, coverage, longevity and capacity of future IoT networks. An efficient relay assignment with proper power allocation and splitting is required to satisfy the network’s QoS requirements. This work considers the resource management problem in decode and forward relay based cooperative IoT network. A realistic mathematical model is proposed for joint user admission, relay assignment, power allocation and splitting ratio selection problem. The optimization problem is a mixed integer non-linear problem (MINLP) whose objective is to maximize the overall sum rate (bps) while satisfying the practical network constraints. Further, an outer approximation algorithm is adopted which provides epsilon-optimal solution to the problem with guaranteed convergence and reasonable complexity. Simulations of the proposed solution are carried out for various network scenarios. The simulation results demonstrate that cooperative communication with diversity achieves a better admission of IoT users and increases not only their individual data rates but also the overall sum rate of an IoT network.

## 1. Introduction

Internet of Things (IoT) is an emerging paradigm which refers to a set of uniquely identifiable devices, capable of communication on a large scale via Internet. The IoT devices perform tasks in a wide range of applications which include smart homes, connected vehicles, industrial automation, environment monitoring, remote asset control, smart hospitals, and many more. One striking application of IoT concept is the wireless sensor network (WSN) in which IoT devices are typically equipped with sensors, controller, wireless transceiver and energy source. These devices have capability of monitoring several physical parameters (e.g., temperature, pressure, humidity, etc.) of their surroundings. In a practical WSN, a massive number of heterogenous sensor devices are deployed over a large geographical area. Each device works autonomously and transmits its data cooperatively by the network to a centralized location for processing or storage.

One of the important challenges to realize massive WSNs is to supply sufficient energy to fulfill their QoS requirements. Mostly, the WSN devices are battery powered with limited resources and deployed at remote locations which lack facility of direct power source or battery replacement. Moreover, the massive data processing and continuous transmission by a WSN device, makes it susceptible to energy depletion. Therefore, improving the energy efficiency of a WSN is mandatory to ensure its long-term and self sustainable operation. Several methods have been proposed in this context, including lightweight communication protocols [1] and smart transceiver designs [2]. Energy harvesting from ambient sources is a promising solution to increase the longevity of WSN devices. For large size applications, exploitation of energy has been maturely done from conventional renewable sources, e.g., solar, wind and geothermal [3]. However, the energy provided by these sources is dependent upon natural conditions and cannot be used in situations where QoS is of prime concern. Moreover, energy harvesters for conventional sources are not compatible with miniature wireless sensor nodes [4]. RF energy harvesting (RF-EH) is an attractive approach to power nodes in emerging WSNs [5]. It provides some key benefits including low cost, always availability and small form factor implementation of energy harvester. An important characteristic of RF-EH is that RF signal allows simultaneous wireless information and power transfer (SWIPT). This allows power constrained devices to harvest energy from RF signal as well as process the useful information contained in it. SWIPT has been successfully applied in a number of low power cellular network scenarios [6,7,8].

Cooperative communication (CC) is a promising technique to enhance the coverage and capacity of wireless networks. A cooperative wireless network consists of relay nodes which assist in information transfer between the source and destination. Typically, the idle users in the network serve as relays and spend their own energy to help active users, leading to decrease in their battery life. Incorporating EH in relays helps to improve the efficiency and reliability of networks with power and QoS constraints. Two commonly used relaying strategies in CC are: (a) amplify and forward (AF); and (b) decode and forward (DF). An AF relay simply amplifies the received signal and retransmits it. On the other hand, a DF relay decodes, re-modulates and re-transmits the received signal. In practical DF-EH relays, energy harvesting and information decoding steps are performed separately using time-switching or power-splitting techniques [9,10]. This is because practical circuits cannot perform energy harvesting and data processing from the same signal simultaneously. Depending upon the scenario, a relay may be able to support simplex, half duplex or full duplex communication in a point-to-point or multi-hop network.

## 2. Literature Review

Significant research effort has been devoted in the domain of resource management in EH cooperative networks. This section discusses some remarkable contributions in this domain. A detailed summary of few recent works is presented in Table 1. The existing works are evaluated on the basis of various characteristics which include network type i.e., single or multi relay, EH capability, support for joint power allocation and user admission, modeling of network’s QoS constraints, nature of optimization problem and proposed solution. In Ref. [11], continuous time and discrete time EH protocols are proposed for AF and DF relay networks. A single source-single relay network model is considered. The work proposes that continuous time EH can be performed on any percentage of block transmission time, whereas in discrete time EH, the whole block is used exclusively for EH or information transfer. A multi-relay cooperative network is considered in Ref. [12] which proposes a greedy algorithm to solve the power allocation for sum rate maximization problem. In Ref. [13], a SWIPT based cooperative network with multiple AF relays is considered for joint relay assignment and power allocation problem. Further, a heuristic is proposed to obtain the solution. In Ref. [14], a non convex optimization problem is formulated for power allocation in a network with QoS constraints and solved with the help of a heuristic. The work in Ref. [15] models the joint relay assignment and power allocation problem as a convex optimization problem and proposes a heuristic to obtain the solution. In Ref. [16], the authors proposed a performance evaluation framework for various relay selection schemes in beacon assisted dual hop cognitive radio networks. In Ref. [17], the authors studied the throughput maximization problem for the orthogonal relay channel with EH source and relay nodes, assuming a deterministic EH model. In Ref. [18], the joint time switching and power allocation optimization problem is investigated for multi-carrier DF relay network with time splitting based relaying. The work in Ref. [19] investigates the maximum transmission rate of PSR/TSR protocols in EH-DF based relay networks. The work considers a single relay network model and formulates the sum rate maximization problem as a convex optimization problem. The work in Ref. [20] investigates the optimal power allocation for two-way half-duplex relaying channel with DF relays. Further, the work solves the throughput maximization problem using subgradient descent algorithm. In Ref. [21], simultaneous wireless power transfer and secure multicasting is considered for DF multi relay network in the presence of multiple energy receivers and eavesdroppers. In Ref. [22], the authors proposed a joint resource allocation for SWIPT based DF relay networks, where the relay adopts a “harvest-then-use” strategy to forward information. To ease the implementation cost, a greedy algorithm for antenna clustering is proposed where the relay antennas are partitioned into two disjoint groups; one for information decoding and the other for energy harvesting. In Ref. [23], a wireless energy harvesting protocol for an underlay cognitive relay network with multiple primary users is proposed. In Ref. [24], SWIPT multi-relay assisted two-hop cooperative network is considered using power splitting DF relays. The convex problem of relay assignment is solved using an interior-point method. The work in Ref. [25] considers a relay assisted network with multiple source-destination pairs and a hybrid relay node. A charge-then-forward protocol is investigated in which the hybrid relay with constant supply first acts as an energy transmitter to charge the sources, and then forwards the information from the sources to their destinations through frequency division multiple access. The optimization problem maximizes the sum rate of all transmission pairs by jointly optimizing the time, frequency and power resources. Further, the problem is solved using the Lagrange duality method. In Ref. [26], the authors studied the joint optimization problem of transmit power and power splitting ratio to maximize the sum rate of cognitive sensor network by considering the interference constraint. In Ref. [27], the sum rate maximization problem is considered for EH MIMO system with underlay spectrum sharing. In Ref. [28], a transmission scheduling scheme is proposed to maximize the packed delivery ratio of multi-terminal cooperative EH networks. Joint achievement of self-backhaul and energy harvesting in small cell networks is studied in Ref. [29]. In Ref. [30], the authors proposed greedy maximal scheduling algorithm for multi-user multi-task computation offloading in green mobile edge cloud computing. The work in Ref. [31] proposes an approach for solving the power and spectrum issues in WSN deployed in hostile environment by harvesting power and spectrum from primary user. Long-term average sum error rate minimization problem for the cooperative network with DF-EH relay node is investigated in Ref. [32]. The energy harvesting and wireless energy transferring networks that were coded over finite field has been studied in Ref. [33], where the energy efficiency was maximized under energy causality and outage probability constraints. The literature summary in Table 1 demonstrates that almost all existing works focus only on one or limited aspects of resource management in EH cooperative networks. In contrast, this work proposes a more realistic mathematical framework which jointly optimizes the user admission, relay assignment, transmit power allocation and selection of power split ratio, and maximizes the overall network sum rate by considering not only the QoS but also practical network constraints. The detailed model is discussed in text that follows.

## 3. System Model

**Figure 1:**
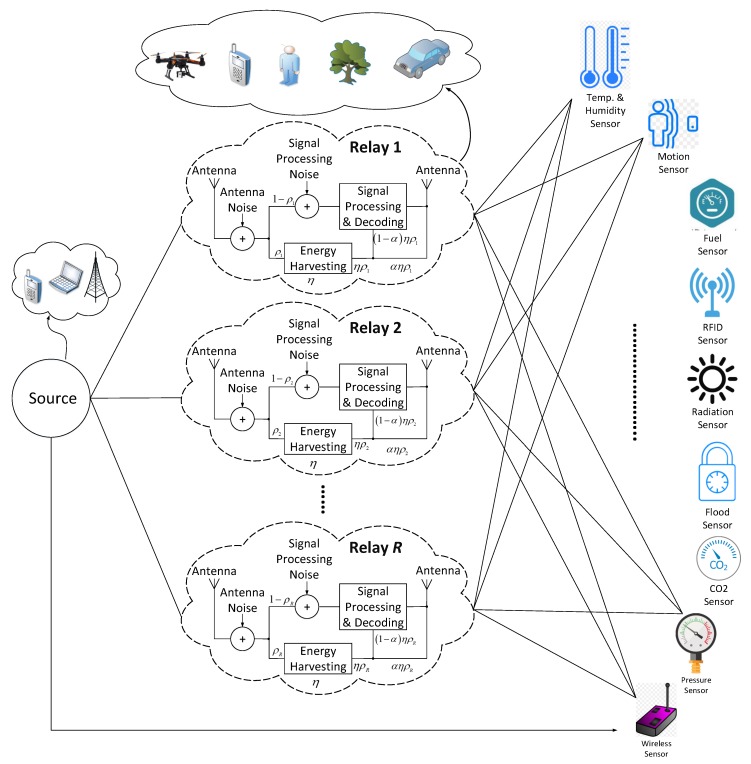
Example scenario of a multi-relay architecture.

This work considers an IoT enabled cooperative WSN scenario. Figure 1 demonstrates an example of such network where a single source node communicates with multiple IoT enabled destination nodes through a network of relay nodes. The source is a SWIPT node, whereas each relay is an energy harvesting DF relay. Practically, any IoT enabled device (e.g., smart phone, fixed or drone base station) can be used to relay information in cooperative communication and may or may not have its own energy source. However, this work assumes that each relay completely relies on energy harvested from the incoming RF signal. A DF relay *i* receives a noise corrupted RF signal with power $P_{i}$ from the source node and splits it into two power components with a ratio $\rho_{i}\in \left[0,1\right]$. One portion of the signal (with power $\rho_{i}P_{i}$) is used for energy harvesting with efficiency η∈[0,1], whereas the other portion (with power $\left(1-\rho_{i}\right)P_{i}$) undergoes signal processing and decoding steps. The energy harvester produces a signal power of $\eta \rho_{i}P_{i}$ at its output. This harvested signal is further split in two power components with a ratio α. One component (i.e., $\left(1-\alpha \right)\eta \rho_{i}P_{i}$) of harvested power is used to energize the signal processing and decoding circuits, whereas the other component (i.e., $\alpha \eta \rho_{i}P_{i}$) is added to the decoded signal for retransmission.

In the network of Figure 1, three cases of communication can be considered: (1) non-cooperative (NC), where the source node communicates with the destination note through a direct link only; (2) cooperative without diversity, where the source communicates with destination node through relay node only; and (3) cooperative with diversity, where the source communicates with destination nodes (in the rest of the paper, the IoT destination nodes are simply called users) using both direct link and relay. For each symbol duration *T* of RF signal, the DF relay harvests energy in the first half slot (of duration $\frac{T}{2}$) and performs retransmission in the second half slot. A cooperative multi-relay scenario offers several advantages which include more network reliability, more admitted IoT users and increasing each user’s individual as well as network’s overall data rate.

Let *K* denote the number of users and *R* denote the number of relays in the cooperative IoT network. The harvested energy at the *r*th relay for the *k*th user frequency band during first half slot T/2 is given as [14],

(1) $$E_{H}^{rk}=\eta_{r}\rho_{r}P_{s}^{k}{\left|h_{sr}\right|}^{2}\frac{T}{2},$$

where $\rho_{r}$ is power splitting ratio at relay *r*, $|h_{sr}|$ is the complex channel gain between source and relay *r* and $\eta_{r}$ is the energy harvesting efficiency of relay *r*. $\alpha_{r}$ portion of harvested energy is transmitted in second half slot T/2. The corresponding transmit power from *r*th relay to *k*th user is

(2) $$\begin{array}{c} \begin{array}{cc} P_{r}^{k} & =\frac{\alpha_{r}E_{H}^{rk}}{T/2}=\frac{\alpha_{r}\eta_{r}\rho_{r}P_{s}^{k}{\left|h_{sr}\right|}^{2}\frac{T}{2}}{T/2}=\alpha_{r}\eta \rho_{r}P_{s}^{k}{\left|h_{sr}\right|}^{2} \end{array} \end{array}$$

The data rate of communication link with CC without diversity from source to *k*th user with energy harvesting DF relay is given as [11],

(3) $$C_{r}^{WoDiv}=\frac{1}{2}\min\;\;\left\{\log_{2}\left(1+\frac{|\left(1-\rho_{r}\right){\left|h_{sr}\right|}^{2}P_{s}^{k}}{\sigma_{sr}^{2}}\right),\log_{2}\left(1+\frac{|h_{rk}{|}^{2}P_{r}^{k}}{\sigma_{rk}^{2}}+\frac{|h_{sk}{|}^{2}P_{s}^{k}}{\sigma_{sk}^{2}}\right)\right\}$$

where $P_{s}^{k}$ is the transmit power from source to user *k*, $\sigma_{sr}^{2}$, $\sigma_{rk}^{2}$ are the noise contributions in channel from source to relay *r* and relay to user *k* respectively. The $\frac{1}{2}$ term indicates the half-duplex mode of transmission. In Equation (3), the first term indicates the rate of channel between the source and relay *r*, whereas the second term indicates the rate of channel between relay *r* and the user *k*. To avoid congestion, the overall transmission rate for user *k* is the minimum of two rates. In the case of CC with diversity, the effective date rate from source to user *k* with maximum ratio combining is given as,

(4) $$C_{k}^{Div}=\frac{1}{2}\log_{2}\left(1+\frac{|h_{sk}{|}^{2}P_{s}^{k}}{\sigma_{sr}^{2}}\right)+C_{r}^{WoDiv}$$

Putting the value of $P_{r}^{k}$ from Equation (2) into Equation (3), Equation (4) becomes,

(5) $$C_{r}^{WoDiv}=\min\;\;\frac{1}{2}\left\{\log_{2}\left(1+\frac{\left(1-\rho_{r}\right){\left|h_{sr}\right|}^{2}P_{s}^{k}}{\sigma_{sr}^{2}}\right),\log_{2}\left(1+\frac{\rho_{r}\eta P_{s}^{k}\alpha_{r}|h_{sr}{|}^{2}{\left|h_{rk}\right|}^{2}}{\sigma_{rk}^{2}}+\frac{|h_{sk}{|}^{2}P_{s}^{k}}{\sigma_{sk}^{2}}\right)\right\}$$

To satisfy the QoS requirements of an energy harvesting cooperative network, the main challenge is efficient user to relay assignment with best possible power allocation and splitting.

### 3.1. Problem Formulation

This work presents a realistic mathematical model for resource management in cooperative IoT network with energy harvesting. The work models the joint relay assignment, source power and power splitting ratio selection problem while considering the practical network constraints. Table 2 summarizes the main notations and symbols used in the formulation. The mathematical model is described as follows.

#### 3.1.1. Given Parameters

1. The total number of IoT users: *K*
2. The number of potential relays: *R*
3. The QoS (data rate) requirement of users: $R_{k}\;\forall k\in \left\{1,\cdots ,K\right\}$
4. The maximum possible transmit power at the source: $P_{s}^{max}$

#### 3.1.2. Parameters to Determine

1. The source power for each IoT user: $P_{s}^{k}\;\forall k\in \left\{1,\cdots ,K\right\}$
2. The power splitting ratio at the relays: $\rho_{r}\;\forall r\in \left\{1,\cdots ,R\right\}$
3. The selected users binary indicator vector y of size *K*. An entry $y_{k}\in y$ is a “1” if user *k* is selected and “0” otherwise
4. The users to relays assignment binary matrix X of dimension K×R. An entry $x_{r}^{k}\in X$ is a “1” if user *k* is assigned to relay *R* and “0” otherwise

#### 3.1.3. Constraints

- Selected IoT device must satisfy the QoS constraint, i.e., minimum data rate
- Total transmit power must be upper bounded by a specified threshold
- Source power for *k*th band should be zero if *k*th IoT user is not selected for transmission
- Power splitting ratio should be zero if relay is not assigned to any IoT user
- Relay assignment constraint, i.e., one relay must be assigned to at most one user at a time
- Power splitting ratio constraint

#### 3.1.4. Objective

- Obtain the best unknown parameters which maximize the network sum rate while satisfying the constraints

Mathematically, the joint user-relay assignment, source power and splitting ratio selection problem is formulated as:

(6) $$\begin{array}{c} \begin{array}{cc} \mathrm{OP}1:\max_{X,\rho ,P_{s},y} & \;\;\;\sum_{k=1}^{K}\left(\frac{1}{2}\log_{2}\left(1+\frac{|h_{sk}{|}^{2}P_{s}^{k}}{\sigma_{sr}^{2}}\right)+\sum_{r=1}^{R}C_{r}^{WoDiv}\right)\\ \mathrm{subject}\;\mathrm{to}: & \\ & C1:\frac{1}{2}\log_{2}\left(1+\frac{|h_{sk}{|}^{2}P_{s}^{k}}{\sigma_{sr}^{2}}\right)+\sum_{r=1}^{R}C_{r}^{WoDiv}\geq y_{k}R_{k}\;,\;\;\forall \;k\\ & C2:\sum_{k=1}^{K}P_{s}^{k}\leq P_{s}^{max}\\ & C3:P_{s}^{k}\leq y_{k}P_{s}^{max},\;\forall r,\;k\\ & C4:\rho_{r}\leq \sum_{k=1}^{K}x_{r}^{k}\;,\;\;\forall \;r,\;k\\ & C5:\sum_{r=1}^{R}x_{r}^{k}\leq 1\;,\;\;\forall \;k\\ & C6:\sum_{k=1}^{K}x_{r}^{k}\leq 1\;,\;\;\forall \;r\\ & C7:x_{r}^{k}\leq y_{k}\;\;\;\forall \;r,\;k\\ & C8:\rho_{r}\in \left[0,1\right],\;x_{r}^{k}\in \left\{0,1\right\},y_{k}\in \left\{0,1\right\}\;P_{s}^{k}\geq 0\;,\;\forall r,\;k \end{array} \end{array}$$

The problem OP1 of Equation (6) is a mixed integer non-linear optimization problem (MINLP) which maximizes the network sum rate while obtaining the best unknown parameters $X,\rho ,P_{s},y$. The overall data rate of the network is the sum of individual rates of all user-relay assignments. This work considers CC with diversity, i.e., an admitted user can be directly connected to the source as well as through the relay. Therefore, the total data rate achieved by the user is the sum of its rates for both links. The optimization problem is formulated considering the practical constraints as well as QoS requirements of the network. In the formulation, according to constraint C1, a user *k* can be admitted (i.e., $y_{k}$ = “1”) only, if it achieves a certain minimum data rate. The minimum transmit rate is considered as QoS requirement in a number of published work [11,14,16,21]. The constraint C2 is the power budget constraint. It states that the total source power (which is equal to the sum of its transmit powers for all admitted users) must be upper bounded by a certain maximum value $P_{s}^{max}$. According to constraint C3, the source can allocate transmit power for an admitted user only. The constraint C4 enforces that a relay *r* can perform energy harvesting (with power splitting ratio $\rho_{r}$ ) only, if it is assigned. The constraints C5, C6 and C7 ensure that one to one relation ship exists between IoT users and relays i.e., no two users can be assigned to a same relay and not two relays can be assigned to a same user. According to constraint C8, the splitting ratio $\rho_{r}$ for a relay *r* must be a real number from the interval [0,1], where “0” value indicates the relay is not assigned to any user and “1” indicates that all power is dedicated for energy harvesting.

Since $C_{r}^{WoDiv}$ is minimum of two functions, we can rewrite the problem in Equation (6) as

(7) $$\begin{array}{cc} \mathrm{OP}2:\max_{X,\rho ,P_{s},y,\Gamma } & \;\;\;\sum_{k=1}^{K}\frac{1}{2}\log_{2}\left(1+\frac{|h_{sk}{|}^{2}P_{s}^{k}}{\sigma_{sr}^{2}}\right)+\sum_{r=1}^{R}\Gamma_{r}^{k}\\ \mathrm{subject}\;\mathrm{to}: & \\ & C9:\frac{1}{2}\log_{2}\left(1+\frac{|h_{sk}{|}^{2}P_{s}^{k}}{\sigma_{sr}^{2}}\right)+\frac{1}{2}\log_{2}\left(1+\frac{\left(1-\rho_{r}\right){\left|h_{sr}\right|}^{2}P_{s}^{k}}{\sigma_{sr}^{2}}\right)\geq y_{k}R_{k}\;,\;\;\forall \;k\\ & C10:\frac{1}{2}\log_{2}\left(1+\frac{|h_{sk}{|}^{2}P_{s}^{k}}{\sigma_{sr}^{2}}\right)+\frac{1}{2}\log_{2}\left(1+\frac{\rho_{r}\eta P_{s}^{k}\alpha |h_{sr}{|}^{2}{\left|h_{rk}\right|}^{2}}{\sigma_{rk}^{2}}+\frac{|h_{sk}{|}^{2}P_{s}^{k}}{\sigma_{sk}^{2}}\right)\geq y_{k}R_{k}\;,\;\;\forall \;k\\ & C11:\frac{1}{2}\log_{2}\left(1+\frac{\left(1-\rho_{r}\right){\left|h_{sr}\right|}^{2}P_{s}^{k}}{\sigma_{sr}^{2}}\right)\geq \Gamma_{r}^{k}\;,\;\;\forall \;r,k\\ & C12:\frac{1}{2}\log_{2}\left(1+\frac{\rho_{r}\eta P_{s}^{k}\alpha |h_{sr}{|}^{2}{\left|h_{rk}\right|}^{2}}{\sigma_{rk}^{2}}+\frac{|h_{sk}{|}^{2}P_{s}^{k}}{\sigma_{sk}^{2}}\right)\geq \Gamma_{r}^{k}\;,\;\;\forall \;r,k\\ & C2-C8\;of\;\mathrm{Equation}\;(6) \end{array}$$

By exploring the structure of the optimization problem, we can observe that the first part of the objective function is concave and second part is minimum of two BiConcave functions. The optimal solution must satisfy the following condition

(8) $$\log_{2}\left(1+\frac{\left(1-\rho_{r}\right){\left|h_{sr}\right|}^{2}P_{s}^{k}}{\sigma_{sr}^{2}}\right)=\log_{2}\left(1+\frac{\rho_{r}\eta P_{s}^{k}\alpha |h_{sr}{|}^{2}{\left|h_{rk}\right|}^{2}}{\sigma_{rk}^{2}}+\frac{|h_{sk}{|}^{2}P_{s}^{k}}{\sigma_{sk}^{2}}\right)$$

or

(9) $$\frac{\left(1-\rho_{r}\right){\left|h_{sr}\right|}^{2}}{\sigma_{sr}^{2}}=\frac{\rho_{r}\eta \alpha |h_{sr}{|}^{2}{\left|h_{rk}\right|}^{2}}{\sigma_{rk}^{2}}+\frac{|h_{sk}{|}^{2}}{\sigma_{sk}^{2}}$$

Solving Equation (9) gives

(10) $$\rho_{r}=\frac{\frac{|h_{sr}{|}^{2}}{\sigma_{sr}^{2}}-\frac{|h_{sk}{|}^{2}}{\sigma_{sk}^{2}}}{\frac{\eta \alpha |h_{sr}{|}^{2}{\left|h_{rk}\right|}^{2}}{\sigma_{rk}^{2}}+\frac{|h_{sr}{|}^{2}}{\sigma_{sr}^{2}}}$$

Taking into account the constraints C3 and C8 of Equation (6), the optimal value of power splitting factor will be

(11) $$\begin{array}{cc} \rho_{r}^{*} & =\left\{\begin{array}{cc} \max\left(\frac{\frac{|h_{sr}{|}^{2}}{\sigma_{sr}^{2}}-\frac{|h_{sk}{|}^{2}}{\sigma_{sk}^{2}}}{\frac{\eta \alpha |h_{sr}{|}^{2}{\left|h_{rk}\right|}^{2}}{\sigma_{rk}^{2}}+\frac{|h_{sr}{|}^{2}}{\sigma_{sr}^{2}}},0\right), & \mathrm{if}\mathrm{the}x_{r}^{k}=1\\ 0, & \mathrm{otherwise}. \end{array}\right. \end{array}$$

**Figure 2:**
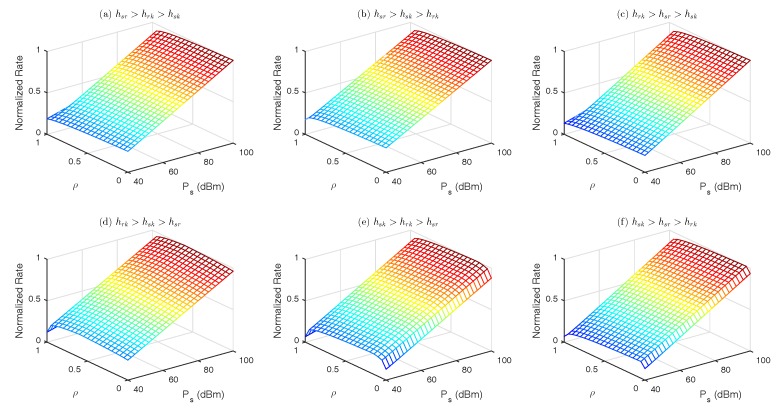
Objective function for different channel scenarios.

The constraint optimization problem OP2 mentioned in Equation (7) is a mixed integer non-linear programming problem which are generally NP-Hard in nature. The main difficulty arises in solving these kind of problems is their combinatorial nature of the domain of discrete variables $X\in {\left\{0,1\right\}}^{NK}$ and $y\in {\left\{0,1\right\}}^{K}$. Any choice of 0 or 1 for the discrete variables X and y results in a non-linear problem on the continuous variables $\rho ,P_{s}$ which can be solved for its best solution. Figure 2 demonstrates a 3D plot of objective function OP2 of Equation (7) for six different cases of channel. For each case, the channel gain is obtained from a Rayleigh distribution. The figure shows that for all cases, the objective function is uni-modal, regular and its local maxima is the global maxima. One may apply brute-force approach to solve this problem but that is computationally expensive. For each discrete realization of $X\in {\left\{0,1\right\}}^{NK}$ and $y\in {\left\{0,1\right\}}^{K}$ variables, the optimizer needs to solve one non-linear programming problem (NLP). The search space for brute force approach is $2^{NK}$, which grows exponentially with the number of IoT devices and relays. Keeping in view the structure of the optimization problem, we apply outer approximation algorithm (OAA) to solve MINLP problem. The proposed OAA gives ϵ- optimal solution with guaranteed convergence. In the next section, we present OAA to solve Equation (7).

## 4. Proposed Approach to a Solution

Since the problem is challenging due to coupling of discrete integer domain with the continuous domain. If we exploit the structure of the problem OP2, it is observed that for discrete realization of variables the problem is a class of convex optimization problem for continuous domain. The proposed outer approximation algorithm works by decomposing the problem mentioned in Equation (7) into a sequence of non-linear subproblems and a sequence of mixed integer problems known as master problems [34,35]. The non-linear subproblem is obtained by fixing the binary integer decision variables $X\in {\left\{0,1\right\}}^{NK}$ and $y\in {\left\{0,1\right\}}^{K}$ in the original MINLP OP2. The non-linear subproblem known as primal problem gives upper bound for the original MINLP problem OP2. The master problem which is the linear approximation of the original MINLP OP2 provides the lower bound for the problem. The algorithm iteratively minimizes the gap between upper bound (primal problem) and lower bound (master problem) till the gap is less than some value ϵ.

First, we define some symbols that represent the short form of the optimization problem. Let $\Gamma^{o}$ and $\Gamma^{c}$ be the objective function and set of constraints in Equation (7), respectively. One can easily verify that for any discrete realization of $X\in {\left\{0,1\right\}}^{NK}$ and $y\in {\left\{0,1\right\}}^{K}$, the objective and constraints in Equation (7) are twice differentiable and the set of continuous variables is convex, compact and non-empty. With these properties of objective function and constraints, the proposed OAA will converge with parameter ϵ in finite number of iterations [35,36]. The proposed OAA is also applicable to non-convex problem but may get stuck in local optimal solution. The primal problem is obtained by fixing X and y variables. At the *i*th iteration of OAA, let the values of integer variable be $X^{i}$ and $y^{i}$. We can write the primal problem as:

(12) $$\begin{array}{c} \begin{array}{cc} \underset{\rho ,P_{s}}{\arg\;\min} & \;\;-\Gamma^{o}\left(X^{i},y^{i},\rho ,P_{s}\right)\\ \mathrm{subject}\;\mathrm{to}:\; & \Gamma^{c}\left(X^{i},y^{i},\rho ,P_{s}\right)\leq 0. \end{array} \end{array}$$

The solution obtained from Equation (12) is used for master problem. The primal problem generates the upper bound on the actual optimization problem. By solving master problem, we get the lower bound and discrete variables for the next iteration. The master problem derived across the solution is provided by the primal problem by applying outer approximation on the objective function $\Gamma^{o}\left(X^{i},y^{i},\rho ,P_{s}\right)$. The master problem is solved in two steps. At the start, a projection of Equation (7) is drawn on to X and y,

(13) $$\begin{array}{c} \begin{array}{cc} \min_{X,y}\;\min_{\rho ,P_{s}} & \;\;-\Gamma^{o}\left(X^{i},y^{i},\rho ,P_{s}\right)\\ \mathrm{subject}\;\mathrm{to}:\; & \Gamma^{c}\left(X^{i},y^{i},\rho ,P_{s}\right)\leq 0. \end{array} \end{array}$$

due to convexity of objective function and constraint, we subsequently linearize Equation (13) around $\left(X^{i},y^{i}\right)$ the functions $\Gamma^{o}\left(X^{i},y^{i},\rho ,P_{s}\right)$ and $\Gamma^{c}\left(X^{i},y^{i},\rho ,P_{s}\right)$ as

(14) ΓoX,y,ρ,Ps≥ΓoXi,yi,ρ,Ps+∇ΓoXi,yi,ρ,PsX-XiY-YiΓcX,y,ρ,Ps≥ΓcXi,yi,ρ,Ps+∇ΓcXi,yi,ρ,PsX-XiY-Yi

with these approximations, the master problem in Equation (13) will be

(15) minX,yminρ,Ps-ΓoXi,yi,ρ,Ps+∇ΓoXi,yi,ρ,PsX-XiY-Yisubjectto:ΓcXi,yi,ρ,Ps+∇ΓcXi,yi,ρ,PsX-XiY-Yi≤0.

**Figure 3:**
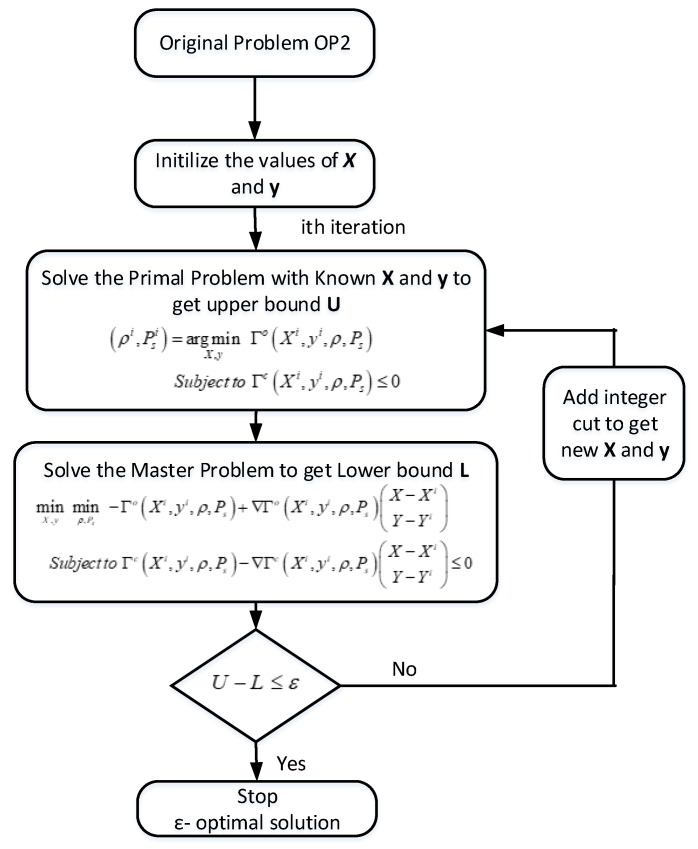
OAA Flow chart.

The solution of the linearized problem mentioned in Equation (15) will give lower bound. The algorithm is iterated until the gap between upper bound from the primal problem and lower bound from the master problem is less thab ϵ. A flow chart of OAA is given in Figure 3.

## 5. Simulation Results

In this section, the simulation results are discussed. The following three scenarios have been simulated and are compared:

1. No cooperation, termed as NC
2. Cooperation with diversity, termed as $C_{\mathrm{Div}}$
3. Cooperation without diversity, termed as $C_{\mathrm{WoDiv}}$

**Figure 4:**
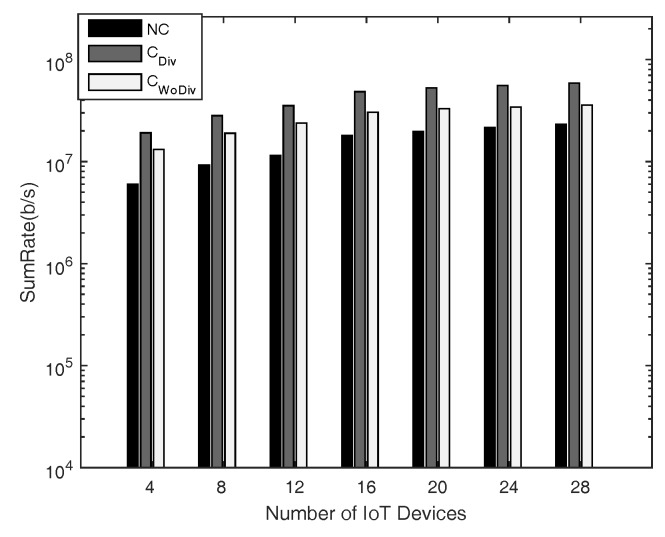
Sum-rate with respect to different number of total devices, with two relays and minimum device data rate of 128 kbps.

**Figure 5:**
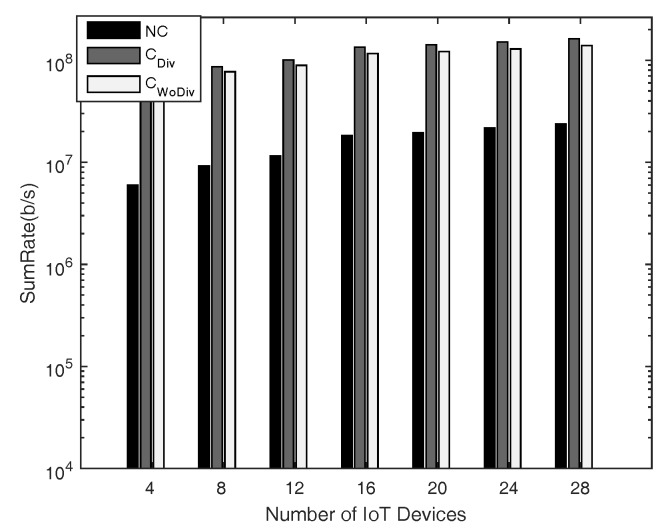
Sum-rate with respect to different number of total devices, with 10 relays and minimum device data rate of 128 kbps.

In Figure 4 and Figure 5, the comparison is given for sum-rate with respect to *K* number of IoT devices, where K={4,8,12,⋯,28}, and *R* number of relays, where R=2 and R=10, respectively. The minimum device rate is 125 kbps in both cases. It is clear that the achieved sum-rate is always better in the case of cooperation in both cases. However, when comparing the two cases of R=2 and R=10 with all other same parameters, it is quite clear that, with more relays, the system is able to satisfy the QoS constraint, i.e., data rate, of more devices. In this way, more devices are admitted and eventually the sum-rate is increased. It is to be noted here that the channel between the devices in both cases discussed above is the same. It is also concluded that more relays provide more paths, hence granting more freedom for the communication. Overall, in all cases, the sum-rate is maximized when diversity is introduced. With more relays the difference between the cooperation and the non-cooperation has also increased because in cooperation more and more devices can be admitted. Therefore, the overall sum-rate can be increased by deploying more relays along with diversity.

**Figure 6:**
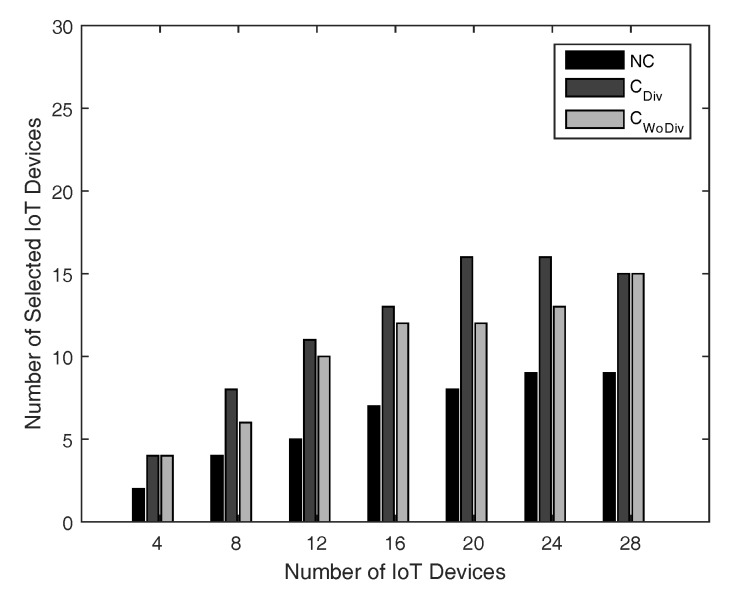
No. of admitted devices with respect to different number of total devices, with two relays and minimum device data rate of 1 Mbps.

**Figure 7:**
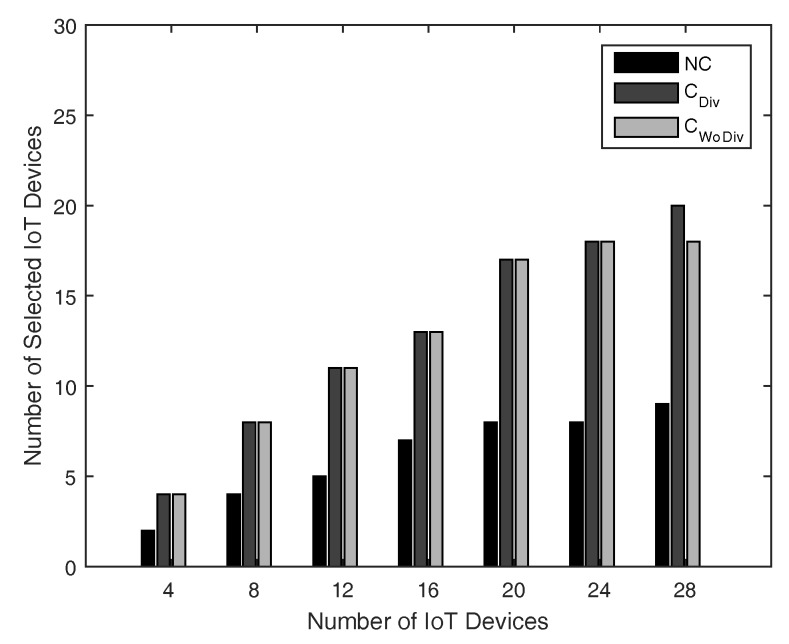
No. of admitted devices with respect to different number of total devices, with 10 relays and minimum device data rate of 1 Mbps.

The number of admitted devices with respect total number of devices is shown in Figure 6 and Figure 7. In both cases, the minimum data rate is 1 Mbps for all devices, whereas the number of relays are 2 and 10 in Figure 6 and Figure 7, respectively. With fewer relays, the QoS constraint (data rate ) for most of the devices could not be satisfied and therefore fewer devices would be admitted. However, as soon as the number of relays are increased, more communication for association will allow more devices to be admitted. Once again, the user admission is better in case of cooperation communication and can be further improved if diversity is also introduced.

**Figure 8:**
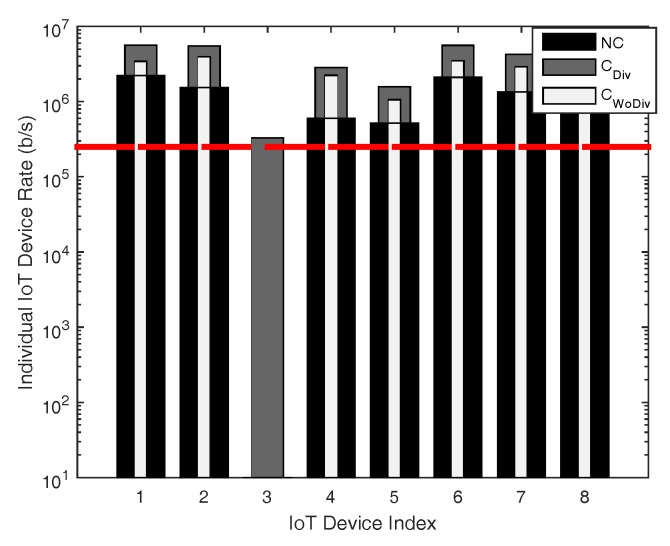
Individual device achieved data rate with respect to different number of total devices, with two relays and minimum device data rate of 250 kbps.

**Figure 9:**
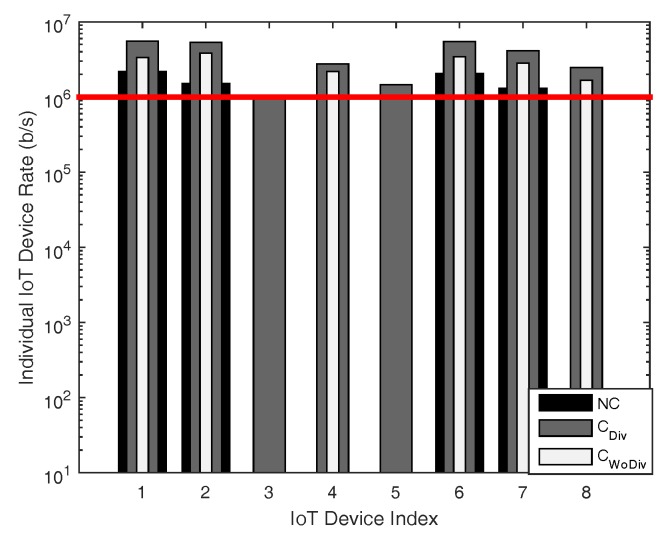
Individual device achieved data rate with respect to different number of total devices, with two relays and minimum device data rate of 1 Mbps.

**Figure 10:**
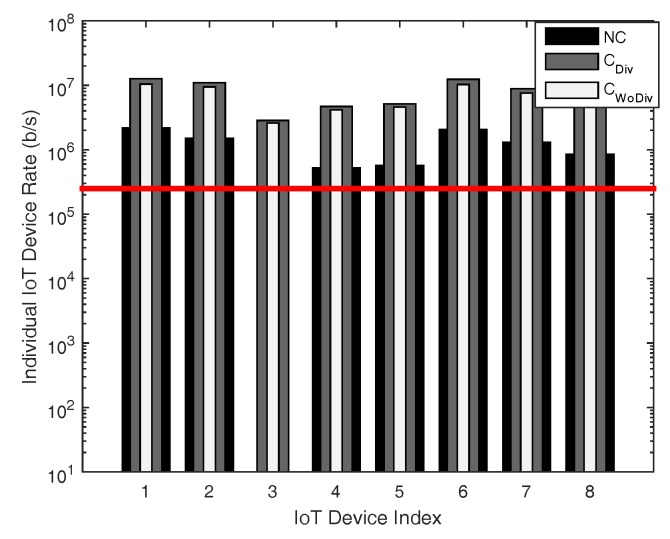
Individual device achieved data rate with respect to different number of total devices, with six relays and minimum device data rate of 250 kbps.

**Figure 11:**
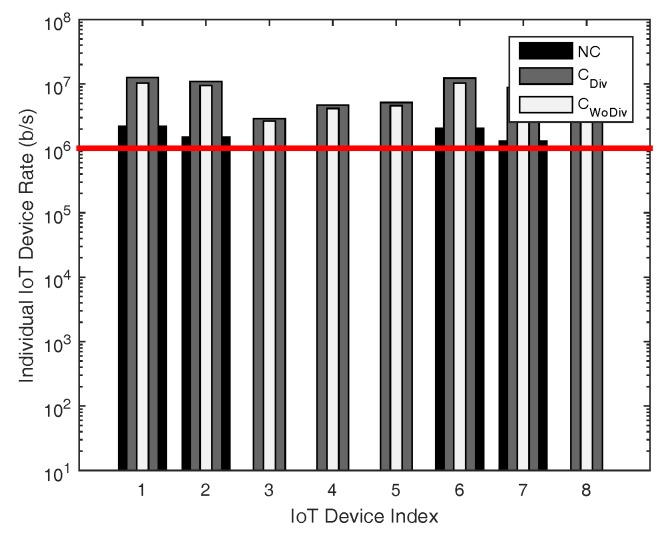
Individual device achieved data rate with respect to different number of total devices, with six relays and minimum device data rate of 1 Mbps.

In Figure 8, Figure 9, Figure 10 and Figure 11, the individual device rate with respect to different number of devices *K*, where K={1,2,3,⋯,8} is studied. In Figure 8 and Figure 9, the total number of relays is two, whereas the target rate is 250 kbps and 1 Mbps, respectively. A device cannot be admitted if the QoS constraint of a device is not satisfied, i.e., the data rate of that device is unreachable. As in both cases the number of relays are equal, it is quite evident that more devices in the case of least data rate should be admitted, as depicted in Figure 8 and Figure 9. As the minimum data rate requirement in Figure 8 is far less than the data rate requirement in Figure 9, the optimizer can distribute the available power in more devices to satisfy their data rate requirement. However, when the minimum data rate requirement is increased from 250 kbps to 1 Mbps in Figure 9, it becomes difficult for the optimizer to distribute the available power among all the devices to satisfy their QoS constraint, which eventually results in the reduction of admitted user. Overall, with less data rate requirement, cooperation with diversity admits more devices.

**Figure 12:**
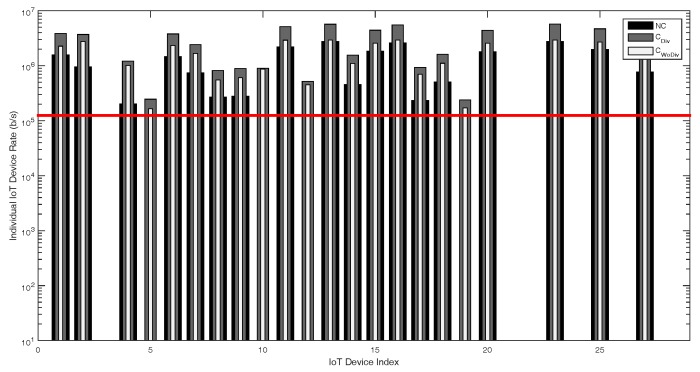
Individual device achieved data rate with respect to different number of total devices, with two relays and minimum device data rate of 125 kbps.

Similarly, in Figure 10 and Figure 11, the individual device’s achieved data rate is presented. In this case, the number of relays is increased from two to six, with same data rate requirement of 250 kbps and 1 Mbps, respectively. As evident, more devices are admitted when the relays are increased. Once again, the same conclusion can be drawn from this phenomenon that, when the relays are increased, the QoS constraint of more devices are satisfied. In Figure 12, individual device rate is once again studied with respect to total number of devices *K*, where, in this case, K={1,2,3,⋯,27}. The total number of relays are 2 and the minimum device rate is 125 kbps. In case of cooperation with and without diversity almost 81%, whereas 66% devices are admitted when there is no cooperation. It is clear that, even if the minimum device rate is set low, with the increase in number of devices, fewer devices would be admitted. From these simulation results, it can be concluded that, to admit more and more devices, more relays have to be deployed and the cooperation should have diversity.

**Figure 13:**
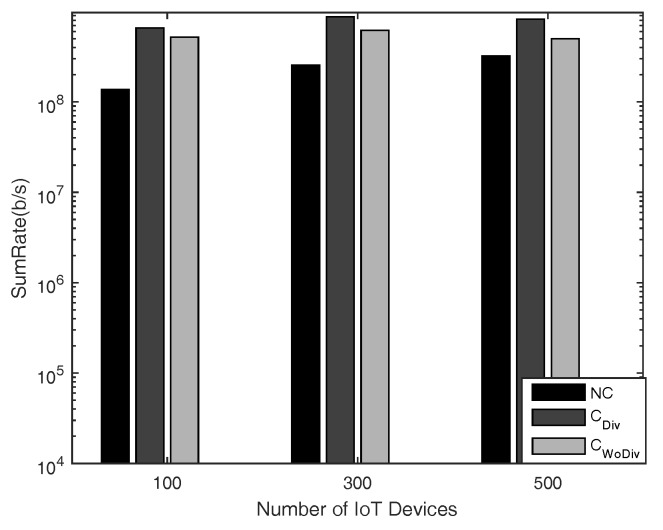
Sum-rate with respect to different number of total devices, with 10 relays and minimum device data rate of 10 kbps.

**Figure 14:**
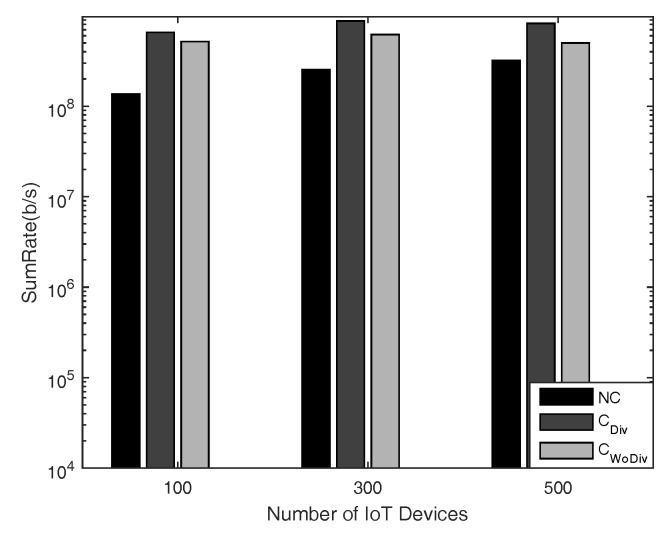
Sum-rate with respect to different number of total devices, with 10 relays and minimum device data rate of 125 kbps.

In general, IoT use cases operate with quite large number of IoT nodes. Keeping this fact of IoT network in mind, simulations have also been carried out for large number of IoT devices, i.e., ranging from 100 devices to 500 devices, as shown in Figure 13 and Figure 14, where the minimum rate is set to 10 kbps and 125 kbps, respectively. It has been concluded above that the cooperation with diversity outperforms in terms of sum-rate and the number of selected devices compared to other two discussed schemes in the case of small number of IoT devices and high data rates. Similarly, in the case many devices and low data rates, the cooperation with diversity is still leading in terms of the metrics discussed above.

In Table 3, the simulation results with multiple scenarios are shown. The different scenarios are created for different number of IoT devices, ranging from 16 to 500 devices, and two different rates, i.e., 10 kbps and 125 kbps. In the table, it is quite clear that, in all scenarios, the cooperation with diversity surpassed the other two schemes with a remarkable margin in terms of achieved sum-rate. Similarly, the user admission in cooperation with diversity is also better than the other two discussed schemes.

## 6. Conclusions

Cooperative communication using energy harvesting relays has emerged as a promising approach to increase the reliability, capacity and efficiency of large area, massive IoT networks. However, to fully exploit the elegant features of this concept, an efficient user-relay assignment scheme is mandatory along with proper allocation and splitting of transmit power. In this work, a resource management problem is addressed in decode and forward relay based cooperative IoT network. First, an accurate mathematical framework is proposed for joint user-relay assignment, transmit power and splitting ratio allocation problem. The proposed formulation maximizes the overall sum rate of the network while considering the practical constraints. Further, an outer approximation algorithm is used to achieve near optimal solution to the problem with reasonable complexity. The accuracy of proposed solution is validated from the simulation results for various practical network scenarios. The results demonstrate that combining cooperative communication with diversity helps to achieve better admission of IoT users and not only increases their individual data rates but also the overall sum rate of the network.

## Figures and Tables

**Table 1:** Literature review of resource management in EH cooperative networks.

Ref.	Relay		EH	Power Alloc.	Admission Control	QoS	Multi-User	Optimization Type	Solution Method
	Single	Multiple							
[11]	✓		✓			✓			Analytical
[12]		✓		✓					Greedy algorithm
[13]		✓	✓	✓				convex	Heuristic
[14]			✓	✓		✓		non-convex	Heuristic
[15]		✓	✓	✓			✓	convex	Heuristic
[16]		✓				✓	✓		Analytical
[17]	✓		✓	✓				convex	Heuristic
[18]	✓		✓	✓				convex	Heuristic
[19]	✓		✓					convex	Analytical
[20]	✓		✓	✓				concave	Iterative subgradient descent method
[21]		✓	✓	✓		✓	✓		Semidefinite relaxation & bisection techniques
[22]	✓		✓	✓				convex	Greedy clustering algorithm
[23]	✓		✓	✓			✓		Analytical
[24]		✓	✓					convex	Interior-point method
[25]	✓		✓	✓			✓	non-convex	Lagrange duality method
[26]	✓		✓	✓				MINLP	Heuristic
[27]			✓				✓		Asymptotic
[28]		✓	✓						Heuristic
[29]			✓	✓			✓	MINLP	Iterative heuristic algorithm
[30]			✓	✓			✓		Greedy maximal scheduling algorithms
[31]	✓		✓						
[32]	✓		✓						
[33]		✓	✓	✓			✓	MINLP	Convex form-based iterative algorithm
**This work**		✓	✓	✓	✓	✓	✓	MINLP	Heuristic

**Table 2:** Notations.

Symbol	Definition
*K*	Total No. of IoT users
*R*	Total No. of relay nodes
$C_{k}^{1}$	Achievable transmit rate at relay
$C_{k}^{2}$	Achievable transmit rate at user
$x_{r}^{k}$	Binary indicator showing relay-user association
$\rho_{r}$	Source power splitting ratio between relay *r* and user *k*
$P_{s}^{k}$	Source power between source and user *k*
$P_{s}^{max}$	Maximum source power
$R_{k}$	Rate for user *k*
$h_{sr}$	Channel gain between source and relay *r*
$h_{rk}$	Channel gain between relay and user *k*
*B*	Bandwidth
$\sigma_{sr}^{2}$	Variance of total noise from source *s* to relay *r*
$\sigma_{sk}^{2}$	Variance of total noise from source *s* to user *k*
$\sigma_{rk}^{2}$	Variance of total noise from relay *r* to user *k*
η	Energy harvesting efficiency
α	Fraction of harvested energy to forward signal

**Table 3:** Simulation results for multiple scenarios, where *K* is number of IoT devices and *R* means number of relays.

Scenario [K, R, Rate (kb/s)]	Sum Rate (Mb/s)			Selected IoT Devices		
	NC	Cdiv	CWoDiv	NC	Cdiv	CWoDiv
[16,10,125]	18.3435	134.292	115.949	13	14	14
[28,10,125]	23.7017	162.82	139.12	18	20	20
[100,10,10]	136.7	655.9	519.2	97	100	100
[100,10,125]	147.1	982.7	835.6	87	100	100
[300,10,10]	254.9	873.6	618.7	294	300	200
[300,10,125]	258.9	1540	1281.1	219	296	295
[500,10,10]	321.4	821.9	500.8	481	500	300
[500,10,125]	317.9	1752.4	1434.5	364	495	495
